# Diversity oriented one-pot three-component sequential synthesis of annulated benzothiazoloquinazolines

**DOI:** 10.1186/2191-2858-2-10

**Published:** 2012-03-02

**Authors:** Mahendra Kumar, Kailash Sharma, Dinesh Kumar Sharma

**Affiliations:** 1 Department of Chemistry, University of Rajasthan, Jaipur 302 055, India

**Keywords:** benzothiazoloquinazolines, one-pot, 2-aminobenzothiazoles, cyclic ketones, thiophene-2-carbaldehyde.

## Abstract

Annulated benzothiazoloquinazolines have been synthesized by a diversity oriented simple and convenient synthesis involving one-pot three-component reaction of substituted 2-aminobenzothiazoles with α-tetralone and aromatic/heteroaromatic aldehydes in ethanol in the presence of catalytic amount of triethylamine. The synthesized compounds have been characterized by their elemental analyses and spectral data.

## Background

The synthesis of fused heterocycles has attracted considerable interest in heterocyclic chemistry as the fusion of biodynamic heterosystems has proved to be a very attractive and useful for the design of new molecular framework of potential drugs with varying pharmacological activities. A major challenge of modern drug discovery is to design highly efficient chemical reaction sequences which provide molecules containing maximum complexity and structural diversity with interesting bioactivities in minimum number of synthetic steps. Recently, multicomponent reactions have attracted attention of chemical and pharmaceutical research and emerged as highly efficient and synthetically useful reactions for the preparation of structurally diverse drug- like heterocyclic compounds.

There has been an increasing interest in the chemistry of quinazolines [1-5] because of being present quinazoline heterocyclic system as a building block in many natural and synthetic products capable of exhibiting a wide variety of biological and pharmacological activities [6]. Quinazolines have been reported to exhibit anti-inflammatory [7], antihypertensive [8], anticancer [9], antitumor [10] and antibacterial [11] activities. Quinazoline and its derivatives have recently been evaluated as antagonist of various biological receptors such as 5-HT_5A _[12] related disease calcitonin gene - related peptide [13] and vasopressin V3 receptors [14]. Benzothiazole has also been an interesting heterocyclic system in drug research on account of significant biological activities of its fused derivatives [15-17]. Thiazoloquinazolines, have shown significant activity against cancer [18]. Thiazoloquinazolines have also been identified as cyclin dependent kinase (CDK) and glycogen synthase kinase (GSK-3) inhibitors [19,20].

## Result and discussion

**Scheme 1:**
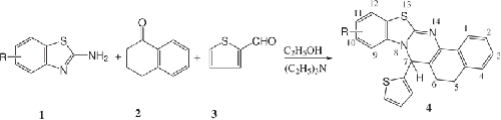


The syntheses of heterocycles with fused heterosystems involve, in most of the cases, multistep synthetic methods which require a large number of synthetic operations including extraction and purification of each individual step. The multistep synthetic methodologies, therefore, led to the synthetic inefficiency with the generation of large amounts of waste. The methods reported in the literature [21,22] for the synthesis of thiazoloquinazolines irrespective of positions of attachment of both the heterocyclic systems involved multisteps in which the formation of thiazole ring before the quinazoline ring induced low subsequent reactivity. Thiazoloquinazolines prepared by reaction of 2-aminobenzylamine with aromatic amine and then with 2-mercaptopropionic acid also involved multistep reaction. However, after detailed literature survey it was observed that there were only limited publications devoted to the synthesis of especially benzothiazoloquinazolines which involved cyclocondensation of 2-aminobenzothiazoles with Mannich bases obtained in the first step as a result of Mannich reaction [23]. The multistep synthesis of benzothiazoloquinazolines suffered from some flaws including low yields, side products and tedious workup. In continuation of our research programme on the synthesis of nitrogen and sulphur containing novel heterocycles [24-31] of pharmaceutical interest and in view of the operational simplicity and intrinsic convergence (atom economy) of multicomponent reactions [32-36] in addition to their potential to introduce considerable structural diversity, we have synthesized annulated benzothiazoloquinazolines incorporating three biodynamic privileged heterosystems by a simple and convenient method involving multicomponent reaction of substituted 2-aminobenzothiazoles with α-tetralone and aromatic/heteroaromatic aldehydes in ethanol in the presence of catalytic amount of triethylamine, wherein 2-aminobenzothiazoles with both endocyclic nitrogen and exocyclic amino group participate as 1,3-binucleophile synthones in the formation of annulated benzothiazoloquinazolines (Scheme 1).

The reaction is believed to proceed to involve Knoevenagal condensation between α-tetralone and aromatic/heteroaromatic aldehyde in the initial step to form α,β- unsaturated ketone which undergoes Michael type addition with the nucleophilic endocyclic nitrogen of 2-aminobenzothiazole under the reaction conditions. The adduct formed is then cyclised intramolecularly with the lose of water molecule to provide annulated benzothiazolo[2,3-*b*] quinazolines. These reactions have taken place in one-flask domino manner and the enone system generated in situ immediately undergoes Michael type addition with 2-aminobenzothiazoles and subsequent cyclization. The possibility of formation of isomeric product involving nucleophilic attack of exocyclic amino group has been completely discarded on the basis of spectral characteristics. Annulated benzothiazoloquinazolines have also been synthesized by a multistep synthesis involving the reaction of substituted 2-aminobenzothiazoles with chalcones obtained by the reaction of α-tetralone with thiophene-2-carbaldehyde/p-methoxybenzaldehyde in the presence of catalytic amount of triethylamine. It has been observed that with the use of chalcones the reaction occurs in two steps relatively in longer time (6-8 h) with the moderate yields (50-60%) of annulated benzothiazoloquinazolines. But when the chalcones are replaced by their synthetic precursors, α-tetralone and thiophene-2-carbaldehyde/p-methoxybenzaldehyde, annulated benzothiazoloquinazolines are obtained with excellent yields (75-85%). The synthesized annulated benzothiazoloquinazolines are presented in table 1.

**Table 1:** Reaction of 2-aminobenzothiazoles with α-tetralone and aromatic/heteroaromatic aldehydes.

2-aminobenzothiazole	α-tetralone	Aldehydes	product	m.p.°C	% yield
				210-212	76
				220-222	80
				205-207	77
				190-192	75
				215-217	80
				227-229	80
				192-194	74
				189-191	75

The structures of the synthesized compounds were confirmed by their elemental analyses and spectral data. Infrared spectra of all the synthesized compounds exhibit an intense unsaturation absorption band in the region 1605-1610 cm^-1 ^(C = C). The absorption band in the region 1620-1654 cm^-1 ^in the IR spectra of all the compounds indicated the presence of C = N bond. Two absorption bands corresponding to asymmetric and symmetric stretching vibrations of -NH_2 _group, which were present in the IR spectra of 2-aminobenzothiazoles, are absent in the IR spectra of the synthesized compounds. ^1^H NMR spectra of the synthesized compounds showed a multiplet in the region δ 7.01-8.08 ppm due to aromatic protons. The singlet appeared in the region δ 5.90-6.80 was assigned to the aliphatic proton (C_7_-H). The singlet observed in the region δ 3.69-3.84 ppm was attributed to the methoxy protons, whereas the methyl protons resonate as a singlet in the region δ 2.34-2.36 ppm in the ^1^H NMR spectra of annulated benzothiazoloquinazolines containing -OCH_3 _and -CH_3 _groups respectively. The multiplets observed in the region δ 2.74-3.23 ppm were assigned to the methylene protons at C-5 and C-6. In the ^13^C NMR spectra of the synthesized annulated benzothiazoloquinazolines, the δ values of most of the carbon atoms of CH_3_, CH_2_, CH, C = C, C = N, C-N, C-O, C-S, C-F and aromatic system could be determined by distinct resonance signals and were found to be in agreement with the proposed structures.

## Conclusion

In conclusion, we have developed a simple and convenient diversity oriented one-pot three-component sequential synthesis for the synthesis of structurally diverse heterocycles, annulated benzothiazoloquinazolines, incorporating medicinally privileged heterosystems.

The present method with its mild reaction conditions and operational simplicity enables the sequential combination of three reactive components; α-tetralone, thiophene-2-carbaldehyde/p-methoxybenzaldehyde and 2-aminobenzothiazole in one-pot and efficiently incorporates structural diversity simply by varying the substituents or by slight structural modification in the components involved in the reaction. This new method has the advantages of higher yields, mild reaction conditions, shorter reaction time, and convenient procedure and can be extended to prepare a library of structurally diverse drug like small heterocyclic molecules.

## Experimental

Melting points were determined on an electric melting point apparatus and are uncorrected. The purity of all the compounds was checked by thin layer chromatography using various non-aqueous solvents. IR spectra (KBr pellets) were recorded on Shimadzu-8400S FT-IR Spectrophotometer. The ^1^H NMR and ^13^C NMR spectra were scanned on Jeol AL 300 FT NMR in CDCl_3 _using TMS as an internal standard. The chemical shifts are expressed as δ ppm.

## Methods

### Procedure for the preparation of 7-thien-2-yl/4'-methoxyphenyl-5, 6-dihydro-7H-benzo [h] benzothiazolo [2, 3-b] quinazolines

To a magnetically stirred solution of thiophene-2-carbaldehyde/p-methoxy benzaldehyde (0.01 mole) and α-tetralone (0.01 mole) in ethanol (20 ml), a catalytic amount of (C_2_H_5_)_3_N was added slowly and further stirred for 30 min. Then 2-aminobenzothiazole (0.01 mole) was added to the reaction mixture with stirring for 2 h and then refluxed for 60-90 min in a round bottom flask fitted with reflux condenser. The progress of the reaction was monitored by TLC. The precipitate formed after the completion of reaction was isolated by filtration. The solid separated out was washed well with ethanol, dried and finally crystallized from ethanol.

### 11-Methyl-7-(thien-2-yl)-5,6-dihydro-7H-benzo[h]benzothiazolo[2, 3-b]quinazoline (4a)

***(4a)***. m.p. 210-212°C; yield: 76%; IR (KBr, cm^-1^): 3100, 2980, 1620, 1605; ^1^H NMR (300 MHz, CDCl_3_) δppm: 7.10-7.68 (m, 10H, Ar-H), 5.86 (s, 1H, C_7_), 3.18-3.23 (m, 2H, CH_2_), 2.93-3.05 (m, 2H, CH_2_), 2.36 (s, 3H, CH_3_); ^13^C NMR (75 MHz, CDCl_3_) δppm: 18.38, 26.98, 28.10, 58.32, 119.43, 122.13, 125.34, 125.53, 126.23,126.56, 126.85, 127.80, 127.32, 127.54, 128.26, 130.20, 132.14, 133.65, 134.12, 137.34, 139.14,143.15, 167.35; Anal. Calcd. for C_23_H_18_N_2_S_2_: C, 71.47; H, 4.69; N, 7.25; Found: C, 73.65; H, 4.99; N, 7.19.

### 11-Methoxy-7-(thien-2-yl)-5,6-dihydro-7H-benzo[h]benzothiazolo [2, 3-b] quinazoline (4b)

***(4b) ***m.p. 220-222°C; yield: 80%, IR (KBr, cm^-1^): 3010-3080, 2975, 1620, 1615; ^1^H NMR (300 MHz, CDCl_3_) δppm: 7.09-7.84 (m, 10H, Ar-H), 5.98 (s, 1H,C_7_-H), δ 3.84 (s, 3H, OCH_3_), 2.96-3.22 (m, 4H, CH_2_, C_5_, and C_6_); ^13^C NMR (75 MHz, CDCl_3_) δppm: 26.9, 28.06, 54.60, 57.20, 115.70, 122.52, 125.29, 125.62, 126.14, 126.45, 127.58, 127.69, 128.08, 129.44, 130.68, 133.12, 133.28, 134.60, 137.20, 139.04, 142.98, 155.61, 167.19 Anal. Calcd. for C_23_H_18_N_2_OS_2_: C, 68.63; H, 4.51; N, 6.96; Found: C, 70.65; H, 4.35; N, 6.99.

### 9,10-Dichloro-7-(thien-2-yl)-5,6-dihydro-7H-benzo[h]benzothiazolo-[2, 3-b]quinazoline (4c)

***(4c) ***m.p. 205-207°C; yield: 77%, IR (KBr, cm^-1^): 3060, 2972, 1622, 1615; ^1^H NMR (300 MHz, CDCl_3_) δppm: 7.08-7.45 (m, 9H, Ar-H), 5.96 (s, 1H,C_7_-H), δ 3.12-3.24 (m, 2H,CH_2_) 2.94-2.96 (m, 2H,CH_2_); ^13^C NMR (75 MHz, CDCl_3_) δppm: 26.96, 28.16, 57.23, 119.92, 120.32, 123.46, 125.13, 125.24, 126.23, 126.52, 127.26, 127.46, 128.13, 130.32, 131.82, 133.65, 134.91, 138.03, 139.45, 143.98, 167.16; Anal. Calcd. for C_22_H_14_Cl_2_N_2_S_2_: C, 59.86; H, 3.20; N, 6.35; Found: C, 62.09; H, 3.10; N, 6.45.

### 11-Fluoro-7-(thien-2-yl)-5,6-dihydro-7H-benzo[h]benzothiazolo-[2, 3-b]quinazoline (4d)

***(4d) ***m.p. 190-192°C; yield: 75%, IR (KBr, cm^-1^): 3075, 2978, 1620, 1610; ^1^H NMR (300 MHz, CDCl_3_) δ ppm: 6.92-7.40 (m, 10H, Ar-H), 5.95 (s, 1H, C_7_-H), δ 3.18-3.23 (m, 2H,CH_2_) 3.01-3.05 (m, 2H, CH_2_); ^13^C NMR (75 MHz, CDCl_3_) δppm: 26.78, 28.13, 58.62, 119.23, 120.35, 122.16, 125.28, 125.69, 126.31, 126.65, 127.58, 127.67, 128.23, 131.98, 133.54, 134.46, 138.42, 139.68, 142.06, 153.72, 170.43; Anal. Calcd. for C_22_H_15_FN_2_S_2_: C, 67.67; H, 3.87; N, 7.17; Found: C, 67.65; H, 3.83; N, 7.13.

### 11-Methyl-7-(4-methoxyphenyl)-5,6-dihydro-7H-benzo[h]benzothiazolo[2, 3-b]quinazoline (4e)

***(4e) ***m.p. 215-217°C; yield: 80%, IR (KBr, cm^-1^): 3045, 2977, 1650, 1610,1275 ^1^H NMR (300 MHz, CDCl_3_) δppm: 7.20-7.54 (m, 11H, Ar-H), 5.90 (s, 1H, C_7_-H), 3.76 (s, 3H, OCH_3_), 2.94-3.01 (m, 2H, CH_2_), 2.80-2.86 (m, 2H, CH_2_), 2.26 (s, 3H, CH_3_); ^13^C NMR (75 MHz, CDCl_3_) δppm: 18.26, 26.98, 28.68, 54.92, 57.23, 113.74, 114.96, 115.26, 123.64, 125.32, 125.76, 126.23, 126.64, 127.19, 127.84, 128.68, 128.96, 130.05, 132.92, 133.30, 134.52, 143.42, 156.39, 169.30; Anal. Calcd. for C_26_H_22_N_2_OS: C, 76.07; H, 5.40; N, 6.82; Found: C, 76.01; H, 5.15; N, 6.92.

### 11-Methoxy-7-(4-methoxyphenyl)-5,6-dihydro-7H-benzo[h]benzothiazolo-[2, 3-b]quinazoline (4f)

***(4f) ***m.p. 227-229°C; yield: 80%, IR (KBr, cm^-1^): 3100, 3080-3050, 1648, 1612; ^1^H NMR (300 MHz, CDCl_3_) δppm: 7.15-7.52 (m, 11H, Ar-H), 5.90 (s, 1H, C_7_-H), δ 3.73 (s, 3H, OCH_3_), 3.69 (s, 3H, OCH_3_), 2.94-3.00 (m, 2H, CH_2_), 2.80-2.86 (m, 2H, CH_2_); ^13^C NMR (75 MHz, CDCl_3_) δppm: 28.26, 28.95, 54.20, 55.09, 56.96, 115.52, 117.52, 119.32, 120.44, 125.18, 126.48, 127.32, 127.47, 127.80, 128.12, 128.76, 133.07, 133.40, 134.23, 136.35, 137.98, 153.62, 158.26, 169.39; Anal. Calcd. for C_26_H_22_N_2_O_2_S: C, 76.2; H, 5.2; N, 6.86; Found: C, 76.07; H, 5.40; N, 6.82.

### 9,10-Dichloro-7-(4-methoxyphenyl)-5,6-dihydro-7H-benzo[h]benzothiazolo[2, 3-b]quinazoline (4g)

***(4g) ***m.p. 192-194°C; yield: 74%, IR (KBr, cm^-1^): 3008-3050, 2972, 1645, 1612, 1270; ^1^H NMR (300 MHz, CDCl_3_) δppm: 7.12-7.54 (m, 10H, Ar-H), 5.92 (s, 1H, C_7_-H), δ 3.73 (s, 3H,OCH_3_), 2.74-2.99 (m, 4H, CH_2 _at C_5 _and C_6_); ^13^C NMR (75 MHz, CDCl_3_) δppm: 26.98, 28.12, 55.08, 58.74, 115.26, 115.35, 119.62, 120.43, 125.63, 127.48, 127.32, 127.58, 128.11, 128.45, 128.69, 130.24, 131.67, 133.40, 133.86, 134.26, 144.53, 155.62, 167.38; Anal. Calcd. for C_25_H_18_Cl_2_N_2_OS: C, 64.49; H, 3.88; N, 6.06; Found: C, 64.52; H, 3.90; N, 6.02.

### 11-Fluoro-7-(4-methoxyphenyl)-5,6-dihydro-7H-benzo[h]benzothiazolo-[2, 3-b]quinazoline (4h)

***(4h) ***m.p. 189-191°C; yield: 75%, IR(KBr, cm^-1^): 3010-3072, 2974, 1648, 1612, 1242; ^1^H NMR (300 MHz, CDCl_3_) δppm: 7.12-7.54 (m, 11H, Ar-H), 5.95 (s, 1H, C_7_-H), δ 3.69 (s, 3H, OCH_3_), 2.82-2.96 (m, 4H, CH_2 _at C_5 _and C_6_); ^13^C NMR (75 MHz, CDCl_3_) δppm: 26.98, 28.56, 55.63, 57.39, 115.47, 118.78, 119.12, 121.45, 125.73, 126.63, 127.32, 127.58, 128.16, 128.73, 128.97, 131.43, 133.26, 133.89, 134.38, 142.19, 153.76, 170.43; Anal. Calcd. for C_25_H_19_FN_2_OS: C, 72.40; H, 4.60; N, 6.72; Found: C, 72.44; H, 4.62; N, 6.76.

## Competing interests

The authors declare that they have no competing interests.
